# Poly[[[diaqua­cobalt(II)]-bis­[*μ_2_*-1,1′-(butane-1,4-di­yl)diimidazole-*κ^2^* *N* ^3^:*N* ^3′^]] dichloride tetra­hydrate]

**DOI:** 10.1107/S1600536809007478

**Published:** 2009-03-06

**Authors:** Yu Su, Yan-Jun Hou, Zhi-Zhong Sun, Guang-Feng Hou, Jin-Sheng Gao

**Affiliations:** a College of Chemistry and Materials Science, Heilongjiang University, Harbin 150080, People’s Republic of China

## Abstract

In the title compound, {[Co(C_10_H_14_N_4_)_2_(H_2_O)_2_]Cl_2_·4H_2_O}_*n*_, the Co^II^ atom and the mid-point of the 1,1′-butane-1,4-diyl­diimidazole ligands lie on inversion centers. The Co^II^ atom is six-coordinated in a slightly distorted octa­hedral environment by four N atoms from four different ligands and by two O atoms from the water mol­ecules. The Co^II^ atoms are bridged by the ligands into a (4,4) net. Adjacent nets are linked to the chloride anions and uncoordinated water mol­ecules *via* O—H⋯Cl and O—H⋯O hydrogen bonds, generating a three-dimensional supra­molecular structure.

## Related literature

For the synthesis of 1,1′-butane-1,4-diyldiimidazole, see: Ma *et al.*(2003 ▶); Yu *et al.* (2008 ▶). For a related Co complex, see: Dong & Zhang (2006 ▶).
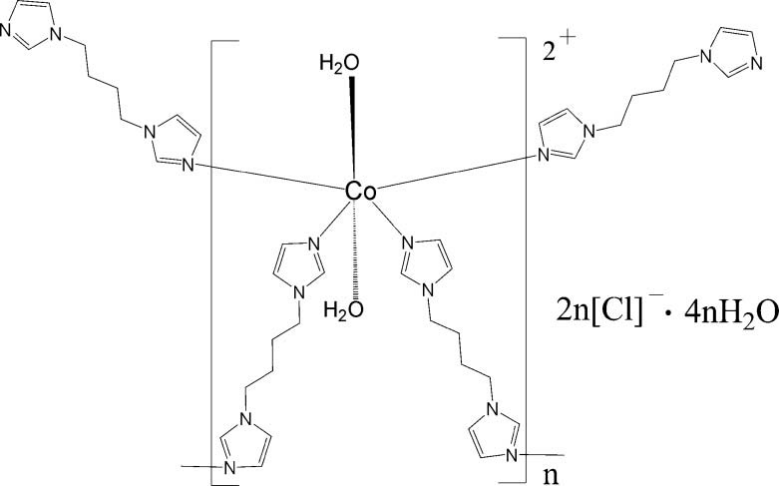


## Experimental

### 

#### Crystal data

[Co(C_10_H_14_N_4_)_2_(H_2_O)_2_]Cl_2_·4H_2_O
*M* *_r_* = 618.43
Triclinic, 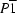

*a* = 7.969 (6) Å
*b* = 9.979 (6) Å
*c* = 10.259 (7) Å
α = 114.97 (2)°
β = 90.83 (3)°
γ = 93.70 (3)°
*V* = 737.3 (8) Å^3^
*Z* = 1
Mo *K*α radiation
μ = 0.81 mm^−1^
*T* = 291 K
0.44 × 0.37 × 0.22 mm

#### Data collection

Rigaku R-AXIS RAPID diffractometer
Absorption correction: multi-scan (*ABSCOR*; Higashi, 1995 ▶) *T*_min_ = 0.718, *T*_max_ = 0.842
7288 measured reflections
3348 independent reflections
3018 reflections with *I* > 2σ(*I*)
*R*_int_ = 0.017

#### Refinement

*R*[*F* ^2^ > 2σ(*F* ^2^)] = 0.028
*wR*(*F* ^2^) = 0.084
*S* = 1.14
3348 reflections
169 parameters
H-atom parameters constrained
Δρ_max_ = 0.33 e Å^−3^
Δρ_min_ = −0.23 e Å^−3^

### 

Data collection: *RAPID-AUTO* (Rigaku, 1998 ▶); cell refinement: *RAPID-AUTO*; data reduction: *CrystalStructure* (Rigaku/MSC, 2002 ▶); program(s) used to solve structure: *SHELXS97* (Sheldrick, 2008 ▶); program(s) used to refine structure: *SHELXL97* (Sheldrick, 2008 ▶); molecular graphics: *SHELXTL* (Sheldrick, 2008 ▶); software used to prepare material for publication: *SHELXL97*.

## Supplementary Material

Crystal structure: contains datablocks global, I. DOI: 10.1107/S1600536809007478/ng2551sup1.cif

Structure factors: contains datablocks I. DOI: 10.1107/S1600536809007478/ng2551Isup2.hkl

Additional supplementary materials:  crystallographic information; 3D view; checkCIF report

## Figures and Tables

**Table 1:** Selected geometric parameters (Å, °)

Co1—N1	2.1265 (18)
Co1—N3	2.1355 (18)
Co1—O1	2.1819 (17)

Symmetry code: (i) 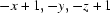.

**Table 2:** Hydrogen-bond geometry (Å, °)

*D*—H⋯*A*	*D*—H	H⋯*A*	*D*⋯*A*	*D*—H⋯*A*
O1—H15⋯O3^ii^	0.85	1.94	2.781 (2)	169
O1—H16⋯Cl1	0.85	2.35	3.1728 (19)	165
O2—H17⋯Cl1^ii^	0.85	2.32	3.172 (2)	176
O2—H18⋯Cl1^iii^	0.85	2.44	3.292 (3)	175
O3—H19⋯O2	0.85	1.99	2.829 (3)	171
O3—H20⋯Cl1	0.85	2.41	3.261 (3)	174

Symmetry codes: (ii) 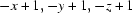; (iii) 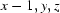.

**Fig. 1.**
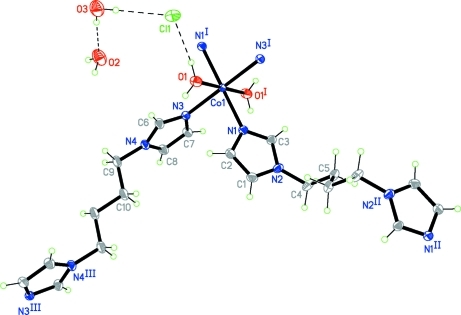
The molecular structure of (I), showing displacement ellipsoids at the 30% probability level for non-H atoms. Dashed lines indicate the hydrogen-bonding interactions [Symmetry code; (I) -x + 1, -y + 1, -z + 1; (II) -x + 2, -y, -z + 2: (III) -x, -y + 1, -z + 2]

**Fig. 2.**
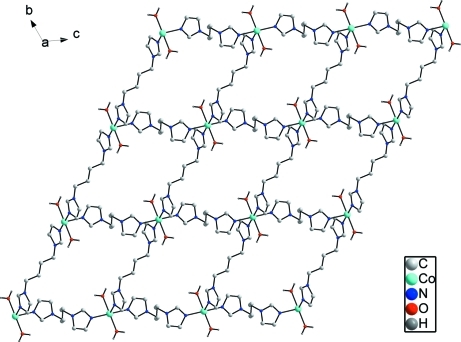
A partial packing view, showing the two-dimensional (4,4)-network. C-bond H atoms have beeb omitted.

**Fig. 3.**
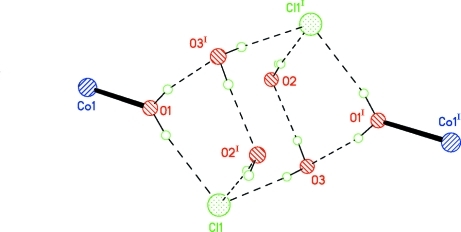
A showing of the hydrogen bonding cluster in I.

**Fig. 4.**
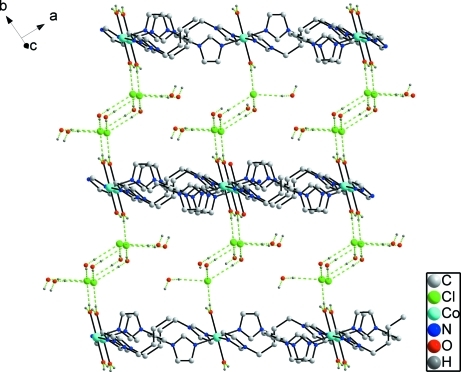
A Partial packing view, shoving the three-dimensional supramolecular structure. Dashed lines indicate the hydrogen-bonding interactions and no involving H atoms have beeb omitted.

## supplementary crystallographic information

### Comment

The *L* molecules as a flexible ligand exhibit a variety of supramolecular aggregation patterns (Ma *et al.*, 2003; Dong *et al.*, 2006; Yu *et al.*, 2008). In this paper, we report the new title compound, (I), synthssized by the reaction of *L* molecules and cobalt dichloride in aqua solution.

In (I), each Co^II^ atom is located on a inversion centre and is six-coordinated in an octahedral environment by four N atoms from four different *L* molecules and two O atoms form the two water molecules (Fig. 1). The Co—N and Co—O distances are normal (Table 1). The Co^II^ atoms are bridged by ligands, generating a two-dimensional (4,4)-network (Fig. 2).

The hydrogen bonding cluster, containing the O—H···Cl and O—H···O hydrogen bonding interaction between the chloride anions, uncoordinated water molecules and the coordinated water molecules (Fig. 3), which linke the adjacent fishnet planes to a three-dimensional supramolecular structure (Fig. 4, Table 2).

### Experimental

*L* was prepared from imidazole and 1,4-dibromobutane in DMSO (Ma *et al.*, 2003). *L* (0.76 g, 4 mmol) and cobalt dichloride (0.51 g, 4 mmol) were dissolved in hot aqua solution (10 ml) to give a clear solution. The resulting solution was allowed to stand in a desiccator at room temperature for a week, red crystals of (I) were obtained.

### Refinement

H atoms bound to C atoms were placed in calculated positions and treated as riding on their parent atoms, with C—H = 0.93 Å (aromatic), C—H = 0.97 Å (methylene), and with *U*_iso_(H) = 1.2*U*_eq_(C). Water H atoms were initially located in a difference Fourier map, but they were treated as riding on their parent atoms with O—H = 0.85 Å and with with *U*_iso_(H) = 1.5*U*_eq_(O).

### Crystal data

**Table:**

[Co(C_10_H_14_N_4_)_2_(H_2_O)_2_]Cl_2_·4H_2_O	*Z* = 1
*M**_r_* = 618.43	*F*(000) = 325
Triclinic, *P*1	*D*_x_ = 1.393 Mg m^−^^3^
Hall symbol: -P 1	Mo *K*α radiation, λ = 0.71073 Å
*a* = 7.969 (6) Å	Cell parameters from 6505 reflections
*b* = 9.979 (6) Å	θ = 3.3–27.5°
*c* = 10.259 (7) Å	µ = 0.81 mm^−^^1^
α = 114.97 (2)°	*T* = 291 K
β = 90.83 (3)°	Block, red
γ = 93.70 (3)°	0.44 × 0.37 × 0.22 mm
*V* = 737.3 (8) Å^3^	

### Data collection

**Table:**

Rigaku R-AXIS RAPID diffractometer	3348 independent reflections
Radiation source: fine-focus sealed tube	3018 reflections with *I* > 2σ(*I*)
graphite	*R*_int_ = 0.017
ω scan	θ_max_ = 27.5°, θ_min_ = 3.3°
Absorption correction: multi-scan (*ABSCOR*; Higashi, 1995)	*h* = −10→10
*T*_min_ = 0.718, *T*_max_ = 0.842	*k* = −12→12
7288 measured reflections	*l* = −13→13

### Refinement

**Table:**

Refinement on *F*^2^	Primary atom site location: structure-invariant direct methods
Least-squares matrix: full	Secondary atom site location: difference Fourier map
*R*[*F*^2^ > 2σ(*F*^2^)] = 0.028	Hydrogen site location: inferred from neighbouring sites
*wR*(*F*^2^) = 0.084	H-atom parameters constrained
*S* = 1.14	*w* = 1/[σ^2^(*F*_o_^2^) + (0.0444*P*)^2^ + 0.1566*P*] where *P* = (*F*_o_^2^ + 2*F*_c_^2^)/3
3348 reflections	(Δ/σ)_max_ < 0.001
169 parameters	Δρ_max_ = 0.33 e Å^−^^3^
0 restraints	Δρ_min_ = −0.22 e Å^−^^3^

### Special details

**Table:**

Geometry. All e.s.d.'s (except the e.s.d. in the dihedral angle between two l.s. planes) are estimated using the full covariance matrix. The cell e.s.d.'s are taken into account individually in the estimation of e.s.d.'s in distances, angles and torsion angles; correlations between e.s.d.'s in cell parameters are only used when they are defined by crystal symmetry. An approximate (isotropic) treatment of cell e.s.d.'s is used for estimating e.s.d.'s involving l.s. planes.
Refinement. Refinement of *F*^2^ against ALL reflections. The weighted *R*-factor *wR* and goodness of fit *S* are based on *F*^2^, conventional *R*-factors *R* are based on *F*, with *F* set to zero for negative *F*^2^. The threshold expression of *F*^2^ > σ(*F*^2^) is used only for calculating *R*-factors(gt) *etc*. and is not relevant to the choice of reflections for refinement. *R*-factors based on *F*^2^ are statistically about twice as large as those based on *F*, and *R*- factors based on ALL data will be even larger.

### Fractional atomic coordinates and isotropic or equivalent isotropic displacement parameters (Å^2^)

**Table:**

	*x*	*y*	*z*	*U*_iso_*/*U*_eq_	
C1	0.6521 (2)	0.1455 (2)	0.94674 (17)	0.0354 (4)	
H1	0.6695	0.2157	1.0416	0.043*	
C2	0.5873 (2)	0.16729 (19)	0.83506 (17)	0.0330 (3)	
H2	0.5516	0.2565	0.8409	0.040*	
C3	0.64306 (19)	−0.06103 (17)	0.75067 (16)	0.0283 (3)	
H3	0.6543	−0.1596	0.6885	0.034*	
C4	0.7646 (2)	−0.0765 (2)	0.9698 (2)	0.0382 (4)	
H4	0.7167	−0.1783	0.9312	0.046*	
H5	0.7386	−0.0288	1.0705	0.046*	
C5	0.9543 (2)	−0.07561 (17)	0.95821 (18)	0.0335 (3)	
H6	0.9958	−0.1443	0.9931	0.040*	
H7	0.9803	−0.1107	0.8576	0.040*	
C6	0.2259 (2)	0.22255 (18)	0.64249 (18)	0.0331 (3)	
H8	0.2873	0.2993	0.6314	0.040*	
C7	0.1451 (2)	0.00888 (18)	0.63070 (18)	0.0325 (3)	
H9	0.1409	−0.0916	0.6093	0.039*	
C8	0.0303 (2)	0.10224 (18)	0.70471 (18)	0.0344 (4)	
H10	−0.0658	0.0784	0.7432	0.041*	
C9	0.0058 (2)	0.3775 (2)	0.7958 (2)	0.0406 (4)	
H11	−0.1151	0.3629	0.7760	0.049*	
H12	0.0495	0.4529	0.7663	0.049*	
C10	0.0423 (3)	0.43001 (19)	0.9552 (2)	0.0438 (4)	
H13	0.1630	0.4487	0.9751	0.053*	
H14	0.0038	0.3522	0.9832	0.053*	
Cl1	0.74821 (7)	0.35621 (5)	0.32791 (5)	0.04818 (14)	
Co1	0.5000	0.0000	0.5000	0.02144 (9)	
N1	0.58238 (16)	0.03688 (14)	0.71136 (13)	0.0274 (3)	
N2	0.68668 (16)	−0.00004 (15)	0.89242 (14)	0.0299 (3)	
N3	0.26947 (15)	0.08534 (14)	0.59164 (13)	0.0265 (3)	
N4	0.08195 (17)	0.23842 (15)	0.71246 (15)	0.0312 (3)	
O1	0.59361 (16)	0.22377 (12)	0.53595 (13)	0.0381 (3)	
H15	0.5827	0.3049	0.6089	0.057*	
H16	0.6273	0.2429	0.4670	0.057*	
O2	0.1615 (2)	0.38041 (17)	0.36469 (18)	0.0668 (5)	
H17	0.1812	0.4490	0.4489	0.100*	
H18	0.0554	0.3700	0.3490	0.100*	
O3	0.4231 (2)	0.49280 (17)	0.24618 (19)	0.0670 (5)	
H19	0.3378	0.4575	0.2734	0.100*	
H20	0.5121	0.4639	0.2693	0.100*	

### Atomic displacement parameters (Å^2^)

**Table:**

	*U*^11^	*U*^22^	*U*^33^	*U*^12^	*U*^13^	*U*^23^
C1	0.0329 (8)	0.0426 (9)	0.0241 (7)	0.0052 (7)	0.0005 (6)	0.0074 (7)
C2	0.0328 (8)	0.0345 (8)	0.0280 (7)	0.0091 (6)	0.0016 (6)	0.0086 (7)
C3	0.0268 (7)	0.0319 (8)	0.0256 (7)	0.0010 (6)	−0.0018 (6)	0.0120 (6)
C4	0.0357 (9)	0.0503 (10)	0.0395 (9)	−0.0060 (7)	−0.0089 (7)	0.0314 (8)
C5	0.0354 (9)	0.0321 (8)	0.0369 (8)	0.0016 (6)	−0.0083 (7)	0.0188 (7)
C6	0.0291 (8)	0.0340 (8)	0.0418 (9)	0.0078 (6)	0.0106 (7)	0.0205 (7)
C7	0.0335 (8)	0.0283 (8)	0.0337 (8)	0.0033 (6)	0.0067 (6)	0.0110 (7)
C8	0.0306 (8)	0.0358 (8)	0.0378 (8)	0.0041 (6)	0.0110 (7)	0.0161 (7)
C9	0.0412 (10)	0.0369 (9)	0.0508 (10)	0.0190 (7)	0.0179 (8)	0.0228 (8)
C10	0.0513 (11)	0.0324 (9)	0.0497 (11)	0.0197 (8)	0.0149 (9)	0.0166 (8)
Cl1	0.0565 (3)	0.0506 (3)	0.0353 (2)	−0.0058 (2)	0.0008 (2)	0.0175 (2)
Co1	0.02117 (15)	0.02402 (15)	0.01902 (14)	0.00459 (10)	0.00162 (10)	0.00862 (11)
N1	0.0255 (6)	0.0329 (7)	0.0226 (6)	0.0051 (5)	0.0004 (5)	0.0104 (5)
N2	0.0247 (6)	0.0420 (7)	0.0260 (6)	0.0001 (5)	−0.0020 (5)	0.0179 (6)
N3	0.0235 (6)	0.0316 (6)	0.0256 (6)	0.0061 (5)	0.0038 (5)	0.0127 (5)
N4	0.0290 (7)	0.0328 (7)	0.0353 (7)	0.0105 (5)	0.0103 (5)	0.0166 (6)
O1	0.0505 (8)	0.0261 (6)	0.0355 (6)	0.0007 (5)	0.0115 (5)	0.0110 (5)
O2	0.0597 (10)	0.0521 (9)	0.0657 (10)	0.0088 (7)	−0.0033 (8)	0.0025 (8)
O3	0.0763 (12)	0.0472 (9)	0.0683 (10)	0.0111 (8)	0.0051 (9)	0.0147 (8)

### Geometric parameters (Å, °)

**Table:**

C1—C2	1.354 (3)	C8—N4	1.363 (2)
C1—N2	1.367 (2)	C8—H10	0.9300
C1—H1	0.9300	C9—N4	1.466 (2)
C2—N1	1.380 (2)	C9—C10	1.508 (3)
C2—H2	0.9300	C9—H11	0.9700
C3—N1	1.319 (2)	C9—H12	0.9700
C3—N2	1.348 (2)	C10—C10^ii^	1.518 (3)
C3—H3	0.9300	C10—H13	0.9700
C4—N2	1.468 (2)	C10—H14	0.9700
C4—C5	1.518 (3)	Co1—N1^iii^	2.1265 (18)
C4—H4	0.9700	Co1—N1	2.1265 (18)
C4—H5	0.9700	Co1—N3	2.1355 (18)
C5—C5^i^	1.513 (3)	Co1—N3^iii^	2.1355 (18)
C5—H6	0.9700	Co1—O1	2.1819 (17)
C5—H7	0.9700	Co1—O1^iii^	2.1819 (17)
C6—N3	1.316 (2)	O1—H15	0.8500
C6—N4	1.345 (2)	O1—H16	0.8501
C6—H8	0.9300	O2—H17	0.8501
C7—C8	1.347 (2)	O2—H18	0.8499
C7—N3	1.378 (2)	O3—H19	0.8500
C7—H9	0.9300	O3—H20	0.8501
			
C2—C1—N2	106.40 (14)	C9—C10—H13	109.1
C2—C1—H1	126.8	C10^ii^—C10—H13	109.1
N2—C1—H1	126.8	C9—C10—H14	109.1
C1—C2—N1	109.55 (16)	C10^ii^—C10—H14	109.1
C1—C2—H2	125.2	H13—C10—H14	107.8
N1—C2—H2	125.2	N1^iii^—Co1—N1	180.0
N1—C3—N2	111.34 (14)	N1^iii^—Co1—N3	93.49 (6)
N1—C3—H3	124.3	N1—Co1—N3	86.51 (6)
N2—C3—H3	124.3	N1^iii^—Co1—N3^iii^	86.51 (6)
N2—C4—C5	112.55 (14)	N1—Co1—N3^iii^	93.49 (6)
N2—C4—H4	109.1	N3—Co1—N3^iii^	180.0
C5—C4—H4	109.1	N1^iii^—Co1—O1	88.40 (6)
N2—C4—H5	109.1	N1—Co1—O1	91.60 (6)
C5—C4—H5	109.1	N3—Co1—O1	88.99 (6)
H4—C4—H5	107.8	N3^iii^—Co1—O1	91.01 (6)
C5^i^—C5—C4	113.60 (19)	N1^iii^—Co1—O1^iii^	91.60 (6)
C5^i^—C5—H6	108.8	N1—Co1—O1^iii^	88.40 (6)
C4—C5—H6	108.8	N3—Co1—O1^iii^	91.01 (6)
C5^i^—C5—H7	108.8	N3^iii^—Co1—O1^iii^	88.99 (6)
C4—C5—H7	108.8	O1—Co1—O1^iii^	180.0
H6—C5—H7	107.7	C3—N1—C2	105.50 (14)
N3—C6—N4	111.80 (15)	C3—N1—Co1	126.67 (11)
N3—C6—H8	124.1	C2—N1—Co1	127.81 (12)
N4—C6—H8	124.1	C3—N2—C1	107.20 (14)
C8—C7—N3	109.45 (15)	C3—N2—C4	125.39 (15)
C8—C7—H9	125.3	C1—N2—C4	127.34 (14)
N3—C7—H9	125.3	C6—N3—C7	105.19 (14)
C7—C8—N4	106.96 (15)	C6—N3—Co1	128.87 (11)
C7—C8—H10	126.5	C7—N3—Co1	125.19 (11)
N4—C8—H10	126.5	C6—N4—C8	106.59 (14)
N4—C9—C10	111.41 (14)	C6—N4—C9	126.86 (15)
N4—C9—H11	109.3	C8—N4—C9	126.17 (14)
C10—C9—H11	109.3	Co1—O1—H15	128.5
N4—C9—H12	109.3	Co1—O1—H16	121.2
C10—C9—H12	109.3	H15—O1—H16	108.9
H11—C9—H12	108.0	H17—O2—H18	106.8
C9—C10—C10^ii^	112.6 (2)	H19—O3—H20	109.6

Symmetry codes: (i) −*x*+2, −*y*, −*z*+2; (ii) −*x*, −*y*+1, −*z*+2; (iii) −*x*+1, −*y*, −*z*+1.

### Hydrogen-bond geometry (Å, °)

**Table:**

*D*—H···*A*	*D*—H	H···*A*	*D*···*A*	*D*—H···*A*
O1—H15···O3^iv^	0.85	1.94	2.781 (2)	169
O1—H16···Cl1	0.85	2.35	3.1728 (19)	165
O2—H17···Cl1^iv^	0.85	2.32	3.172 (2)	176
O2—H18···Cl1^v^	0.85	2.44	3.292 (3)	175
O3—H19···O2	0.85	1.99	2.829 (3)	171
O3—H20···Cl1	0.85	2.41	3.261 (3)	174

Symmetry codes: (iv) −*x*+1, −*y*+1, −*z*+1; (v) *x*−1, *y*, *z*.
